# Collaboration between Psychiatrists and Other Allied Medical Specialists for the Treatment of Delusional Disorders

**DOI:** 10.3390/healthcare10091729

**Published:** 2022-09-08

**Authors:** Alexandre González-Rodríguez, José Antonio Monreal, Mentxu Natividad, Mary V. Seeman

**Affiliations:** 1 Department of Mental Health, Mutua Terrassa University Hospital, 5 Dr. Robert Square, 08221 Terrassa, Spain; 2 Centro de Investigación Biomédica en Red de Salud Mental (CIBERSAM), University of Barcelona, 08221 Terrassa, Spain; 3 Institut de Neurociències, Universitat Autònoma de Barcelona (UAB), 08221 Terrassa, Spain; 4 Department of Psychiatry, University of Toronto, 605 260 Heath Street West, Toronto, ON M5P 3L6, Canada

**Keywords:** delusional disorder, psychosis, paranoia, collaboration, specialties

## Abstract

Background: There is increasing evidence that individuals with psychosis are at increased risk for cardiovascular disease, diabetes, metabolic syndrome, and several other medical comorbidities. In delusional disorder (DD), this is particularly so because of the relatively late onset age. Aims: The aim of this narrative review is to synthesize the literature on the necessity for medical collaboration between psychiatrists and other specialists. Methods: A non-systematic narrative review was carried out of papers addressing referrals and cooperation among specialists in the care of DD patients. Results: Psychiatrists, the primary care providers for DD patients, depend on neurology to assess cognitive defects and rule out organic sources of delusions. Neurologists rely on psychiatry to help with patient adherence to treatment and the management of psychotropic drug side effects. Psychiatrists require ophthalmology/otolaryngology to treat sensory deficits that often precede delusions; reciprocally, psychiatric consults can help in instances of functional sensory impairment. Close collaboration with dermatologists is essential for treating delusional parasitosis and dysmorphophobia to ensure timely referrals to psychiatry. Conclusions: This review offers many other examples from the literature of the extent of overlap among medical specialties in the evaluation and effective treatment of DD. Optimal patient care requires close collaboration among specialties.

## 1. Introduction

Delusional disorder (DD) is diagnosed by the presence of one or more delusions for a month or longer in individuals who do not fulfil the diagnostic criteria for schizophrenia, affective illness, or primary substance use disorder [1]. The current Diagnostic and Statistical Manual for Mental Disorders, fifth edition (DSM-5) adds the stipulation that delusional disturbances are not attributable to the effects of a medical condition or substance use and do not meet criteria for other mental disorders such as obsessive-compulsive disorder [1].

DD is classified into seven types according to the content of the delusions. The DSM-5 groups these into erotomanic, grandiose, jealous, persecutory, somatic, mixed, and unspecified types [1]. There is often psychiatric comorbidity, the most common being depression, followed by anxiety [2,3].

In schizophrenia and other psychotic disorders, recent evidence has underscored premature mortality when compared to the general population [4]; this has been attributed partly to suicide but mainly to an increased risk of physical health multimorbidity across the psychosis spectrum. An extra risk of metabolic abnormalities has been associated with the use of antipsychotic medications, but occurs in antipsychotic-naïve patients as well [5]. The cardiovascular risk seems to be higher with second-generation antipsychotic (SGA) treatment than with first-generation (FGA) drugs. SGA use has been correlated with weight gain, type 2 diabetes, high blood pressure, and dyslipidemia [6]. Furthermore, several studies have confirmed increased mortality from breast, colon, and lung cancer in patients with schizophrenia (the disorder that has attracted most academic attention), although relative incidence rates remain controversial [7].

In a recent study in patients with a psychosis diagnosis, the strongest comorbid associations were with hearing disturbances and visual impairment, with asthma, diabetes, angina, and arthritis coming next [4]. The relationship between psychosis and comorbid physical illness is complex; among strategies aimed at reducing the burden of physical comorbidity, early detection, and early treatment rank first [8].

The need for early, effective treatment has led to calls for improvements in the collaboration between psychiatry and other medical specialties, particularly neurology [9].

In the context of DD, comorbid medical illnesses have been investigated infrequently. There have been no studies demonstrating the effectiveness of close collaboration between psychiatric experts and other allied medical specialties for this condition. Although a schizophrenia-related disorder, DD differs in many ways from schizophrenia [1]. Because the onset age is relatively late, the risk of medical comorbidities is especially high. In addition, many of the delusions seen in DD involve somatic themes, making accurate causal diagnosis difficult. Patients are frequently seen by medical specialists first, thus delaying early treatment. For all these reasons, close collaboration among specialties is particularly important in DD to improve clinical outcomes.

### Aims

The aim of this selective narrative review is to synthesize the literature on delusional disorder (DD) and the need for medical collaboration. Specifically, in pursuit of integrative and multidisciplinary approaches to the health needs of individuals with DD, the review searches the scientific literature for evidence that close collaboration is essential for timely and effective care of this population, and that this collaboration may improve clinical outcomes.

We address the ways in which neurologists, ophthalmologists, otolaryngologists, dermatologists, pain and sleep specialists, gastroenterologists, cardiologists, and pharmacologists can help psychiatrists who care for patients with DD and, reciprocally, how psychiatrists can assist their colleagues in the care of patients with cognitive deficits, sensory impairment, functional skin, sleep, gastrointestinal, and cardiac symptoms, as well as intractable pain. We are particularly interested in patients with DD because this condition has been understudied, and because it emerges at older ages, at which time patients almost always suffer from significant comorbid medical conditions. Delusional beliefs often involve body organs, so patients visit a variety of medical specialists before referrals are made to psychiatry. Finally, the standard treatment of DD consists of antipsychotics and antidepressants, both of which induce a variety of somatic side effects that often require medical attention.

## 2. Methods

Electronic searches were conducted through the PubMed database for papers referring in their titles or abstracts to specific medical specialties, such as neurology, ophthalmology, otorhinolaryngology, dermatology, pain specialists, gastroenterology, sleep experts, cardiology, and pharmacology. Gynecology and oncology services were not considered, as they had been previously reviewed in this context by our team [10].

The following search terms were used: neurology OR neurologists OR ophthalmology OR ophthalmologists OR blindness OR “hearing impairment” OR “hearing loss” OR otorhinolaryngology OR otorhinolaryngologists OR deafness OR dermatology OR dermatologists OR pain OR gastroenterology OR gastroenterologists OR sleep OR cardiology OR cardiologists OR pharmacology OR pharmacologists AND (“delusional disorder”).

Papers were included if they met the following inclusion criteria: (1) study participants were DD patients who fulfilled DSM or ICD criteria, (2) the paper included a report on the interaction between psychiatry and another medical specialty in the treatment of patients with DD, and (3) the language was English, Spanish, German, or French.

Reference lists from the included studies were screened to add potentially relevant papers in the field not found in the PubMed database. Although we targeted papers published in the last decade (2012–2022), classic papers frequently cited were also included.

**Figure 1:**
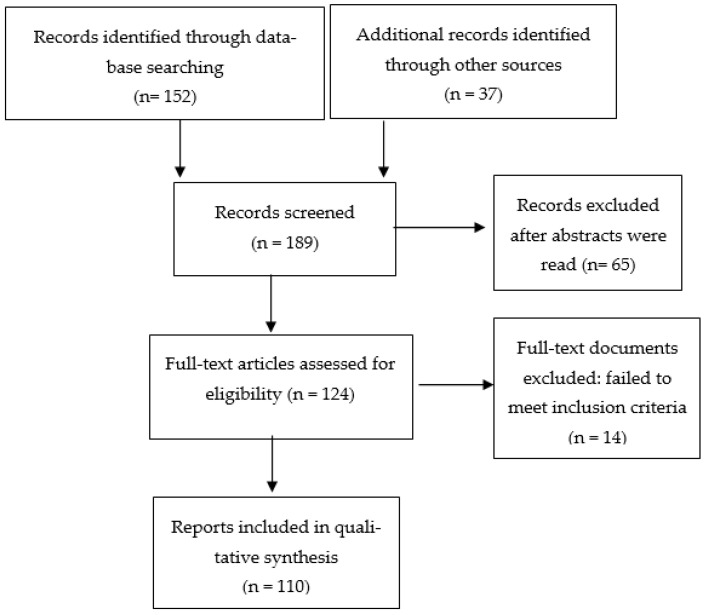
Flow diagram of the included studies.

The screening and selection processes were undertaken by the AGR and MVS. Although several hundred titles and abstracts were scanned, most of them were excluded, as they referred exclusively to patients with schizophrenia. A total of 189 records were screened (PubMed: 152; other sources: 37), from which we selected 110 final studies that were relevant to our aims. Figure 1 shows the screening and selection procedures and the retained studies.

## 3. Collaboration with Neurologists

There are several theoretical reasons why close collaboration between psychiatrists and neurologists would benefit patients with DD. Many DD patients are reported to have cognitive deficits that require specialized investigation [11]. Should such patients first be seen by neurology, they are rapidly referred to psychiatry, often without a thorough neurological workup, an example of diagnostic overshadowing, the incorrect attribution of all a person’s symptoms to a primary psychiatric illness [12]. A thorough neurological assessment and brain scan, if indicated, is important because it can sometimes point to a primary cause of delusions and can predict treatment response and prognosis. The latter is critical for the targeted psychoeducation and support required for patients and family members.

### 3.1. Delusional Disorder Can Precede a Diagnosis of Dementia

The diagnosis of DD with or without depressive symptoms can sometimes precede the onset of Alzheimer’s disease (AD) [13], and the occurrence of delusional parasitosis is sometimes seen as an early symptom of Parkinson’s disease [14]. Cognitive impairment is considered one of the non-motor, usually later, symptoms of Parkinson’s disease (PD), and recent investigations have emphasized that people with PD eventually show an extensive spectrum of cognitive dysfunctions, extensive in terms of severity and also in terms of their morphological basis [15].

Oh and collaborators [16] reported the case of a 75-year-old woman who presented with a main complaint of itching and a previous history of dermatological lesions. She was diagnosed by psychiatry as suffering from delusional parasitosis, but 12 months later, she presented with the following motor symptoms: bradykinesia, hypomimia, hand tremor, and mild rigidity. This case illustrates the fact that an acute onset of delusional parasitosis can be an early symptom of Parkinson’s disease, as can other psychiatric symptoms, such as depression and anxiety.

An older study retrospectively investigated medical records from 188 patients with AD and compared them with 80 patients free of dementia. The results showed that unipolar depression and paranoid disorders were the two most frequent psychiatric morbidities associated with a subsequent diagnosis of AD [13]. This is in line with a study conducted by Leionen and colleagues [14], who followed 150 patients admitted to a psychogeriatric unit over a 1-year period. From a total of 135 patients with full follow-up data, 24 were diagnosed with major depressive disorder and 18 with DD. Within the group of patients with DD, five (28%) developed a dementing disorder over the succeeding 10 years, which is double the incidence rate of dementia in the general population of the same age.

Many patients presenting with DD are known to show cognitive defects [11], which can worsen over the course of treatment, an example of treatment contributing to morbidity. Psychotropic drugs, especially in seniors (who may be taking multiple drugs for different indications), increase the speed of cognitive decline [16,17,18], which makes periodic neurological and neuropsychological assessments and brain imaging critically important [19].

### 3.2. Cognitive Decline Predicts Poor Response to Psychotropic Medications

Because of the treatment complexity, psychiatrists need neuropsychologists, neurologists, psychopharmacologists, and rehabilitation specialists to help with effective detection and management of cognitive decline.

DD is considered a schizophrenia-related disease, and comparisons between DD and schizophrenia are often made. As part of the Roscommon Family Study, Kendler and Walsh [20] investigated the specific characteristics of schizophreniform disorder, DD, psychotic disorders not otherwise specified, schizophrenia, and affective illnesses. They found a strong association between DD and alcoholism, an association that could contribute to cognitive decline in this population, and that merits both neurological investigation and referral to an alcohol abstention program.

A recent study by Louhija and collaborators [21] investigated the association between cognitive decline and brain atrophy in elderly patients with a first episode of psychosis (FEP), many of whom received an eventual diagnosis of DD. This research team used both magnetic resonance imaging (MRI) and computed tomography (CT); the cognitive performance of study participants was evaluated with the cognitive test battery from the Consortium to Establish a Registry for Alzheimer’s Disease. A total of 85 patients with FEP aged 60 or over, and with onset of psychotic symptoms of less than a year’s duration was included. The vast majority of patients presented signs of cortical atrophy, such as those seen in the early stages of cognitive decline, suggesting that brain atrophy and cognition should be routinely evaluated in elderly patients with FEP. The same research group explored the incidence, specific diagnosis, and structural brain changes in 107 patients with FEP aged over 60 years [22]. Brain atrophy was more frequent in patients diagnosed with psychosis secondary to a medical condition than in patients with schizophrenia, DD, or psychotic depression. FEP at a late age, thus appears, very frequently, to be secondary to degenerative brain changes. The results of this study point, once again, to the need for close collaboration between psychiatrists and neurologists.

Cognitive decline has been associated with a poor response to psychotropic medications in patients with DD. González-Rodríguez et al. [23] conducted a recent review of DD in old age and concluded that cognitive defects in elderly DD patients were potentially associated with poor pharmacological response. This conclusion awaits confirmation by further research. A case report of a 70-year-old woman with delusional parasitosis and cognitive decline [24], followed in an outpatient Partial Psychiatric Hospitalization Program (PPHP), is consistent with this view. Microvascular ischemic changes and cortical atrophy were associated with a poor response to treatment with the antipsychotic, olanzapine, and the antidepressant venlafaxine.

It is possible that brain atrophy and cognitive decline correlate with nonresponse to DD treatment for several reasons—the patient may not be able to remember to take prescribed medication, the medication may, in itself or in interaction with other drugs, be exacerbating poor cognition, or the brain changes may be such that the drugs are not reaching their brain targets. All three factors may be operative.

To sum up, psychiatry needs neurology to help with cognitive issues in DD patients. Neurologists also need psychiatry to help ensure patient adherence to a treatment regimen, to assist with family counseling, and to ascertain and manage psychiatric drug side effects [25,26,27,28].

## 4. Collaboration with Ophthalmology and Otolaryngology

As a consequence of the relatively late age of onset of DD, not only are neurological disorders and various medical conditions comorbid with DD, but so are sensory impairments.

Almeida and collaborators [28] carried out a longitudinal cohort study of a community-representative sample of 38,173 men aged 65 to 85 years. The Western Australian Data Linkage System was used to identify the presence of hearing loss in patients with psychotic disorders, as defined by different versions of the International Classifications of Diseases (ICD). From the total sample, 1442 (3.8%) initially presented with a diagnosis of hearing loss and 464 (1.2%) with psychosis. Those with hearing loss incurred a two-fold higher risk of incident psychosis than those without hearing loss. This suggests that early preventive interventions by otolaryngology may help to reduce psychoses such as DD in this age group. A previous systematic review and meta-analyses also supported the association between psychosis risk and hearing impairment [29]. This research team found an association between hearing impairment and several domains of psychotic symptoms: hallucinations, delusions, and delirium. The review concluded that early assessment and treatment of hearing loss in patients with psychosis is helpful in reducing psychotic symptoms.

In DD populations, Porras-Segovia and colleagues [30] investigated the prevalence of blindness and hearing loss as part of an Andalusian DD case-register study (DelirAnda). The total sample of 1452 patients, all fulfilling DSM-5 criteria for DD, showed a 7.4% prevalence of sensory deficits: blindness (3.5%) and hearing loss (3.9%). This is in agreement with previous research by de Portugal and collaborators [31], who carried out a cross-sectional study of 86 outpatients fulfilling the DSM-IV-TR criteria for DD. Sensory deficits, psychopathological assessments, and other psychosocial risk factors were evaluated, and a 5.7% prevalence of sensory deficits was found. Both of these studies show a prevalence of sensory deficits that is significantly higher than that seen in the general population. An earlier five-year retrospective study on partial deafness and partial blindness in elderly populations [32] showed similar results.

Thakkar et al. reported the case of a 58-year-old man complaining about a 12-month history of burrowing sensation in his eyelids that was attributed by psychiatry to delusional infestation [33]. The patient was subsequently diagnosed with blepharitis and received appropriate treatment but was lost to follow-up. In the conclusion to the report, the authors emphasized the seriousness of a mistaken diagnosis because, in ophthalmology, prompt effective treatment is needed to avoid serious consequences, such as blindness. The ability to consult rapidly on equivocal complaints serves important preventive goals. There is classic literature on the ease of development of paranoid delusions in blind and deaf populations. Case-specific illustrations of patients who became blind and developed persecutory delusions and delusions of jealousy can be found in the writings of Spanish psychiatrist, Sanchís-Banús [34]. There is also modern literature on sensory impairments and delusion formation [35].

Shoham and collaborators [35] carried out a cross-sectional study investigating the association between self-reported hearing and visual impairments with psychotic symptoms in the 2014 UK Adult Psychiatric Morbidity Survey. Psychotic symptoms and social function were strongly correlated with moderate visual impairment and a severe degree of hearing loss. Such findings support a significant etiological association between visual/hearing impairment and psychosis mediated by social isolation. To determine whether their findings were consistent with those of others, the same research group carried out a systematic review and meta-analysis of the topic [36]. The evidence for the association with psychosis proved strongest for visual impairment [37].

Over the past 15 years, there has been an exponential increase in the number of publications hypothesizing that altered retinal structure, function, and vasculature play a part in the origins of psychotic phenomena [38]. The deafferentation theory, a decrease in the threshold for activation in the brain leading to an imbalance between excitatory and inhibitory brain networks, is thought to underlie this relationship [39]. The social isolation that follows sensory impairment is seen as increasing the risk of psychosis, particularly hallucinations. Psychiatrists need the assistance of ophthalmology and otolaryngology, as well as of social rehabilitation specialists, to diagnose, treat, and prevent further deterioration of sensory impairments and to help integrate DD patients with such impairment into a supportive community.

In summary, psychiatrists treating DD need assistance with the sensory impairments of their patients, and ophthalmology/otolaryngology need psychiatric expertise to sort out sensory phenomena that are delusional or caused by antipsychotic treatment or migraine or psychological factors [40].

## 5. Collaboration with Dermatologists

Historically, psychiatry was first welcomed into the medical fold when Austrian psychiatrist Wagner-Jauregg became interested in 3rd stage syphilis (the stage at which spirochetes reached the brain and caused delusions) and received the Nobel prize (1927) for treating what was then called ‘general paresis of the insane’ with malaria inoculation [41,42]. Syphilis, at that time, was the domain of dermatology, so this is a famous instance where psychiatry helped dermatology while, at the same time, establishing itself as a specialty worthy of entering the medical community. Malaria treatment also causes delusions [43], introducing a problem often faced by medicine—that it is sometimes difficult to distinguish between the initial disease and the adverse effects of treating the disease.

Nowadays, a frequent disorder that demands the care of both psychiatrists and dermatologists is delusional parasitosis (also called delusional infestation or Ekbom syndrome) [44]. This is a somatic subtype of DD characterized by the conviction that one is infected by a parasite or other infectious agent, usually through the skin but sometimes also through the gastrointestinal tract [45]. The role of gastroenterologists will be discussed later in this review.

There have been decades of reports about delusional patients with self-perceived skin conditions who first present to dermatologists, internists, and plastic surgeons [46] and are only much later referred to psychiatry. The prevalence of delusional parasitosis increases with age. At midlife, during the period of DD onset, close collaboration between dermatologists and psychiatrists has been acknowledged as crucial [47].

A retrospective study analyzed clinical features in patients with delusional infestations who were admitted to a dermatology ward over a period of two decades [47]. Twenty-one patients were included. Primary DD was diagnosed in 76.2% of the total sample, followed by shared DD (folie à deux) and DD secondary to medical conditions. Only 33.3% of the patients attended scheduled follow-up appointments, and the vast majority of those who did were treated with risperidone (61.9%), an antipsychotic drug. The authors concluded that the effective management of delusional parasitosis remains a major challenge and requires the co-operation of dermatologists and psychiatrists.

A recent multicenter retrospective study recruited 20 patients (median age: 54) with delusional parasitosis from three infectious disease services [48]. Ten patients received antiparasitic treatment, and 8 received a form of psychotropic medication. Only 3 of the 20 achieved a total remission of symptoms, while 9 experienced persistent delusional symptoms. Treatment adherence was judged to be poor, and the recommendation was made that this condition require a multidisciplinary approach.

Concerned about the observation that delusional parasitosis leads to self-induced cutaneous lesions, Torales and collaborators [49] conducted a review of the topic with the aim of updating clinical aspects and treatment strategies for both dermatologists and psychiatrists. Their conclusion was that differential diagnosis, which includes medical conditions and substance use disorders, is crucial to effective treatment, outcome, and prognosis. Similar findings come from tropical medicine services. Todd and collaborators [50] reviewed patients over a 5-year period who attended a clinic staffed by experts in infectious disease, tropical medicine, and psychiatry. From a total sample of 75 patients, 52 were diagnosed with delusional infestation. At follow-up, 61% reported significant improvement in symptoms. The results confirmed the need for collaborative care by experts from different specialties.

In a dermatology service, many patients receive psychiatric treatment not only for DD but also for depression, obsessive-compulsive disorder, and anxiety [51]. Women are particularly vulnerable to the comorbidity of psychotic syndromes with a variety of skin and hair diseases [52]. This is especially true for women with postmenopausal onsets of DD [53], which suggests an added collaboration with endocrinology.

Several studies have reported the positive effects of antidepressants in patients with DD, including patients with delusional parasitosis [54]. However, the vast majority of patients currently receive antipsychotic medications (although patients are extremely reluctant to take them because they firmly believe that they suffer from an infestation and that it is not “in their mind”) [55]. More recently, McPhie and Kirchhof [56] carried out a systematic review of studies examining the effectiveness of typical and atypical antipsychotic medications for primary delusional infestation (both generations of antipsychotics, typical (pimozide was the most commonly reported) and atypical (risperidone was the most common) had favorable outcomes, but the conclusion of this review was that there was a lack of data to strongly support the clinical utility of antipsychotic treatment. The problem could well be one of adherence. In earlier years, it was the custom to treat delusional disorders with an antidepressant that had antipsychotic properties, such as clomipramine, which has been extensively used in the treatment of body dysmorphic disorders and other related delusional themes [57,58]. Because of the stigma associated with psychosis, it is easier to persuade patients with DD to take antidepressants regularly than antipsychotics.

In summary, collaboration between dermatologists and psychiatrists is necessary to improve clinical outcomes in patients suffering not only from delusional parasitosis but also from other types of delusions referable to skin [59], dysmorphophobia involving skin [60], and skin conditions such as acne and hirsutism [61], which are side effects of antipsychotic treatment. The management of these disorders usually implies the need for a multidisciplinary approach, often integrating infectious disease, immunology, and pharmacology services to optimize treatment and improve adherence. Psychodermatology services, such as those described by Seale et al. [61], are a major step in the right direction.

## 6. Collaboration with Pain Specialists

Chronic pain (rheumatic pains, referred pain, fibromyalgia, neuropathies) induces the development of delusional explanations for the pain, but pain can also result from primary delusions [62]. Burning Mouth Syndrome and oral cenesthopathy are specific examples of clinical pain conditions that require collaboration between psychiatrists and pain specialists [63].

A recent study investigated the etiology of oral cenesthopathy (OC) (often categorized as DD somatic type) based on evidence that involvement between the central nervous system and the peripheral nervous system can cause abnormal orofacial sensations [64]. Forty-eight patients with OC were recruited during a 3-year period (2016–2019). Magnetic resonance imaging evaluated the presence of neurovascular contact, and the Oral Dysesthesia Rating Scale was used to assess oral sensations and impairments. The root entry and exit zone of the trigeminal nerve is where the nerve contacts arterial or venous channels, and this contact has been considered a potential source of pain. Such neurovascular contact (NVC) was observed in about half of the patients. Contrary to this hypothesis, OC patients without NVC presented more complex oral symptoms and a significantly higher severity of consequent dysfunction than did patients with NVC. The conclusion was that pain in OC patients was complex in origin, suggesting the possibility of a psychogenic contribution to etiology.

OC has often been considered treatment-resistant and accompanied by major functional impairment. However, Uezato et al. [65] reported a case with a complete response after a modified course of electroconvulsive therapy. Single-photon emission computed tomography revealed abnormal cerebral blood flow before and after treatment. These findings are in line with a classical report by Wada and collaborators, who described a patient with DD somatic-type pain disorder who showed reduced regional cerebral blood flow in the temporal and parietal lobes [66]. The patient was a 78-year-old woman treated with clomipramine 20–100 mg/day. The pain responded only partially, but the patient’s delusions about the pain responded completely.

Karakus and Bulut [63] reported a case of overlapping OC delusions and burning mouth syndrome in an elderly woman. OC symptoms responded to a combination of low-dose aripiprazole and psychoeducation-based cognitive therapy. The quality of life was significantly improved. The conclusion was that both disorders could occur simultaneously, and that multidisciplinary treatment was essential.

To conclude, chronic pain conditions are notoriously difficult to treat and require psychosocial support for patients and family members, especially in the particular context of DD. Psychiatric and psychological skills in individual and family therapy are essential for pain clinic personnel [67], as are the provision of both pharmacological and non-pharmacological treatment [68].

## 7. Collaboration with Gastroenterologists

In cases of delusion referable to the gastrointestinal tract, and also in functional gastrointestinal disease, a collaboration between gastroenterologists and psychiatrists results in improved care for patients.

Tabbalat and collaborators reported two cases of gastrointestinal delusional parasitosis presenting as folie à deux [69]. The first patient was a 58-year-old woman with a medical history of chronic Lyme disease, who, for 2 years, had complained of parasites in her stool. She was taking an herbal supplement recommended by a naturopathic physician. Evidently distressed by the result of her visit to psychiatry, 2 days later, the patient committed suicide. Shortly afterwards, a close friend, the partner in the delusional folie à deux, came to the clinic with her own liquid stool samples and photographs showing elongated shapes in the stool. Ova and parasite tests proved to be negative, as were esophago-gastro-duodenoscopy and colonoscopy tests.

Liver injury with severe coagulopathy secondary to marasmus was observed in a patient with DD somatic type [70]. Acute and severe liver injury occurs due to significant self-induced weight loss, which is the result of self-induced caloric restriction [70]. Treatment was successful, and the liver returned to normal.

A similar report by Geka and collaborators [71] described the case of a 40-year-old woman with DD somatic type who presented with severe weight loss; her body mass index was less than 12. The patient was convinced that she had a severe gastroenterological disease. She attended many gastroenterologists and underwent many laboratory examinations, with all the results being negative. Treatment with olanzapine was found to be effective.

Inflammatory phenomena are considered part of the pathophysiology of both chronic gastrointestinal disorders and chronic psychoses [72,73]. The interplay between gastrointestinal symptoms and mental health disorders has been widely discussed in relation to several disorders [72]. Major histocompatibility complex genes are associated with psychosis, celiac disease, and other gastrointestinal inflammatory diseases [74,75]. With respect to DD, there are studies comparing DD and schizophrenia with respect to the presence of human leukocyte antigen (HLA) class I alleles [76]. One hundred patients with DD and 50 with schizophrenia were compared with a group of healthy volunteers. The HLA-A*03 gene was associated with DD and paranoid schizophrenia, suggesting a shared susceptibility to both disorders. This confirmed the preliminary results of an earlier study by the same group [77].

Chronic illness, such as functional gastrointestinal disorder, is hard to treat and, like pain, lends itself to the development of a delusion that one is being specifically targeted by malevolent forces. This sequence can also take place in the opposite direction. Antipsychotic treatment of DD can trigger gastrointestinal symptoms [78]. Close collaboration between the two specialties benefits both gastrointestinal and psychiatric patients.

## 8. Collaboration with Sleep Specialists

Sleep disorders have been frequently reported in patients with psychosis, including DD [79,80]. Distressing delusional beliefs often haunt patients at night, either preventing sleep or populating nightmares.

Insomnia is the most frequent sleep disorder found in patients with DD [79], and it is associated with an aggravation of paranoid and cognitive symptoms, as well as suicidal ideation. Insomnia and other sleep problems are difficult to address and often require the expertise of sleep specialists.

Narcolepsy—a chronic disorder characterized by sudden attacks of daytime sleep and drowsiness—has been associated with psychotic features hypothetically because of shared immune irregularity [81]. Ten cases of type 1 narcolepsy with comorbid chronic psychosis (one of which was DD) were recruited for the assessment of HLA Class I and II typing. The included patients suffered from type 1 narcolepsy, which is characterized by low–undetectable cerebrospinal fluid Hcrt-1 levels, loss of hypocretin neurons, and is considered an autoimmune disorder. The investigators speculated that narcolepsy therapy may play a role in triggering psychotic symptoms, although changing the narcolepsy medication did not relieve psychosis. There was some response to antipsychotic treatment in these patients, but it was variable. There was no evidence of antibody-mediated autoimmunity or new HLA associations that differed from those of narcolepsy alone. The conclusion of this study was that the combination of narcolepsy and psychosis constitutes a considerable therapeutic challenge.

Restless leg syndrome (RLS), in association with sleep disturbance, has been described in a patient with DD. Basu and collaborators [82] reported the case of a 38-year-old woman suffering from DD and RLS who had been treated with olanzapine and whose RLS improved after treatment with risperidone. RLS is significantly more common in women than in men [83], but no consistent difference in sex prevalence has been found in DD [79].

Obstructive sleep apnea (OSA) is more common in men, but it has been reported in women with psychosis, especially those who are both postmenopausal and obese [84]. Several factors have been identified as potentially increasing the risk for OSA: family history, smoking, alcohol use, and use of antipsychotic medications. Treatment with Continuous Positive Airway Pressure (CPAP) is recommended because it improves sleep parameters and cardiovascular and cognitive outcomes and reduces mortality [85]. OSA should be considered in DD patients who present with negative symptoms (apathy, avolition), since these types of symptoms are relatively rare in DD [86].

Sleep disorders occurring in patients with DD deserve the same specialist care as psychosis-free patients who experience problems related to sleep. Sleep disturbances are often associated with psychosis [87]. A sleep clinic within a psychiatric service was described over 40 years ago [88] and continues to be advocated, though not widely adopted [89].

## 9. Collaboration with Cardiologists

Cardiovascular risk factors are not rare in patients with chronic psychoses, one major one being type 2 diabetes [90]. Diabetes is significantly more prevalent in individuals with psychosis than in the general population for many reasons. These include the use of antipsychotic medication, adverse social circumstances, and genetic loading [90].

Foley and collaborators carried out an observational study on a national sample of 1642 individuals with psychosis recruited through the 2010 Australian National Survey of Psychosis [91]. All fulfilled the ICD-10 criteria for psychotic illnesses, including 92 patients diagnosed with DD. A family history of diabetes was associated with a family history of schizophrenia in the patients with psychosis, no matter the precise diagnosis. The conclusion was that psychosis and diabetes share familial risk factors.

Eriksson and colleagues [92] reported on a study in which they recruited 932 consecutive outpatients with severe mental illness over a 7-year period. The aim was to determine the prevalence of cardiovascular risk factors and treatment with statins and make comparisons with 5580 controls from a population-based survey. Nineteen percent of the sample was diagnosed with DD or psychosis ‘not otherwise specified’. The mean age of the patients was 47, and most were men (65%). Only 4% of the patients received statins. In those with hypertension, only 26% were treated with statins. The rate of cigarette smoking was 44%, compared to 18% in the controls. The evidence was that despite the high frequency of cardiovascular disease in the patients, very few were prescribed statins, an example, yet again, of diagnostic overshadowing. The same research group [93] studied 731 consecutive outpatients with psychosis and compared them with the same 5580 controls. The mean waist circumference for males was 106 cm in patients and 94 cm in controls; for females, it was 97 cm (patients) and 85 cm (controls). Mean fasting glucose for males was 5.8 mmol/L (patients) and 5.2 mmol/L (controls) and for females, 5.6 mmol/L (patients) and 4.8 mmol/L (controls). All relevant factors were controlled. The investigation team concluded that psychosis per se is a cardiovascular risk factor, independent of the more traditional risk factors, such as age and smoking history.

The main risk may be antipsychotic treatment, which increases weight and cholesterol, as well as the chance of diabetes and metabolic syndrome. Some antipsychotics are pro-arrhythmic. Some, such as clozapine, induce cardiomyopathy independently of dose [94].

Cardiologists need to collaborate with psychiatrists when they manage cardiac complaints that are primarily due to panic and anxiety states [95,96,97,98]. A study by Hadlandsmyth and collaborators investigated the frequency of co-morbid anxiety and mood disorders, and acute fear in persons experiencing chest pain [95]. The patients were examined at assessment and one year later. Anxiety and fear about the meaning of chest pain were associated with health care utilization at the time of cardiac evaluation, and fear was still predictive of health care utilization after 1 year.

The assessment of psychopathological symptoms and biographical circumstances seems crucial to accurately interpreting cardiac symptoms, such as pain or arrhythmia [99]. The identification and provision of therapy for stressful past experiences is helpful in the management of such patients [96,97,98].

## 10. Collaboration with Pharmacologists

The side effects of many drugs and interactions among drugs cause body system disturbances that lend themselves to delusions. For instance, many antipsychotics used in the treatment of DD cause hyperprolactinemia. The symptoms of hyperprolactinemia (amenorrhea, galactorrhea) mimic the symptoms of pregnancy and, in vulnerable women, readily lead to delusional pregnancy [100], even in postmenopausal women [101].

In the context of DD in old age, Arbus and collaborators carried out the SAGE (Schizophrenia AGEd), an observational and cross-sectional study focused on the prescription of antipsychotics in elderly patients [102]. From a total sample of 930 patients, 20.8% had been given a DD diagnosis. Forty-six percent of the total sample received atypical antipsychotics, 36.2% typical antipsychotics, and 17.8% a combination of both; 34% had cardiovascular disorders potentially attributable to the use of antipsychotics. Adverse effects are common in elderly populations because liver and renal functions are progressively impaired with age, so elimination is impaired. Although psychiatrists know how to deal with the side effects of the drugs they prescribe, they do not always know about the interactions that can arise with other drugs the patient is taking. Pharmacists can inform patients about the potential for interactions, and pharmacologists can explain why they occur.

They can also help with the process of monitoring antipsychotic levels, an important strategy that can identify potential reasons for poor response and uncover medication nonadherence, a common problem when treating DD patients [103,104,105].

Strauss and collaborators reported the case of a 37-year-old woman with DD treated with risperidone [106]. High plasma levels suggest a potential metabolic disorder, particularly an unusual genetic polymorphism in the CYP2D6 gene. This led to genetic testing, which revealed a heterozygous mutation in the gene that made the patient a poor metabolizer of risperidone. Such tests are important to access when patients fail to respond to treatment. Very few studies have investigated the clinical utility of pharmacogenetic testing of antipsychotics in the context of DD. However, studies of patients with schizophrenia-related disorders have confirmed that this is a useful practice. For instance, Arranz and collaborators [107] evaluated the clinical benefits of pharmacogenetic intervention in the individualization of antipsychotic treatment in schizophrenia. The investigators compared a group of patients whose cytochrome P450 (CYP) pharmacogenetics was determined prior to antipsychotic treatment (*n* = 123) and a comparison group of patients treated without this information (*n* = 167). Patients treated with clozapine who received pharmacogenetics testing showed a significant advantage in drug effectiveness compared to those not tested, suggesting that this is a useful intervention. Other similar studies have also confirmed that patient outcomes improve following pharmacogenetic testing when using both antipsychotics and antidepressants [108], particularly when CYP2D6 and CYP2C19 enzymes are involved in the drug’s metabolism.

The use of long-acting antipsychotic medications is not common in DD but has been described [109] in a longitudinal observational study with a 6-month follow-up that included 45 DD outpatients. The sample was divided into two groups according to the presence or absence of non-prominent hallucinations (relatively rare in DD). Patients receiving long-acting injectable antipsychotics (LOA) were found to have fewer negative symptoms. LOAs are potentially important in conditions such as DD, where adherence to oral medications is poor. Pharmacologists can help advise on timing and incremental doses for switching from oral drugs to LOAs.

In summary, pharmacologists can help psychiatrists identify patterns of antipsychotic metabolism and prevent drug–drug interactions that cause side effects [78] and health problems [110]. Psychiatrists can help pharmacologists by asking pertinent questions and reporting unusual drug reactions that lead to research that results in safer and more effective therapeutic drugs.

Table 1 summarizes why is important close collaboration between psychiatry and other allied specialties.

## 11. Discussion

There are many reasons why close collaboration between psychiatrists and other medical specialists improves health outcomes [111]. This is especially important in our patient group of interest, individuals diagnosed with the understudied and under reported condition of DD. These individuals develop a serious psychotic illness relatively late in life when symptoms can overlap with those of incipient dementing processes. The help of a neurologist to evaluate cognition and obtain and assess brain scans is critical to good care. The age of onset of DD also overlaps with the onset of sensory impairments, which require the expertise of eye doctors and ear, nose, and throat specialists. The social isolation that results from such impairment fosters the development of delusions. Patients with somatic forms of DD, such as delusional parasitosis, often come to psychiatry after having first sought help from dermatologists, plastic surgeons, gastroenterologists, internists, and pain specialists. The somatic type of DD is difficult to treat, and close collaboration among the services involved is crucial for the patient. The side effects of antipsychotic medications used to treat DD also require meaningful collaboration with cardiologists and pharmacologists.

Collaboration comes in many forms. Collaborative services exist, such as psychodermatology, in which psychiatrists work together with other specialists to improve clinical outcomes [112]. A second option is a consultation liaison service that facilitates quick referrals among specialties [113,114,115]. A third potential model considers shared care with nurse practitioners or family physicians. The development of primary care is important and is needed to implement collaborations among specialties [112,113]. Integrated care has been shown to work [116].

## 12. Conclusions

This narrative review describes the collaborative needs of psychiatrists treating patients with delusional disorders. Comprehensive collaborative treatment goes beyond the specialties reviewed here, but this paper begins to illustrate the necessity of liaison among specialties and its potential for improving diagnosis and care and preventing mishaps, delays, and unwarranted complications.

## Figures and Tables

**Table 1:** Reasons for the importance of close collaboration between psychiatry and other medical specialties.

Specialty	How Psychiatry Can Help Other Specialties	How Other Doctors Can Help Psychiatry
Neurology	DD can precede the onset of Parkinson’s disease and other dementias	Investigation of cognitive defects and brain structure helps predict Rx response
Ophthalmology and Otorhinolaryngology	Sensory impairments May have psychological bases	Rx of deafness and blindness reduces DD symptoms
Dermatology	Skin disease may be self-inflicted	Early referral to psychiatry improves outcome
Pain specialty	Chronic pain benefits from psychiatric Rx	Specialized pain Rx helps somatic type DD
Gastroenterology	Psychosis and inflammatory disease often co-exist	DD may show evidence of G.I. inflammation and auto-immunity
Sleep Units	Rx of psychotic symptoms & changes in antipsychotic regimens improve sleep disorders	Sleep apnoea needs to be ruled out in DD patients with negative symptoms
Cardiology	Trauma may precede chest pain and arrhythmias	Antipsychotic selection may need cardiological input
Pharmacology	Reporting adverse drug effects spurs the synthesis of better drugs	Experts help in testing for harmful drug-drug interactions

## Data Availability

The data presented in this review are available on request from the corresponding author.
